# Place vs. Pocketbook: Associations of Area‐Level and Individual‐Level Income on Oral Cavity Cancer Late‐Stage Diagnosis

**DOI:** 10.1002/cam4.71238

**Published:** 2025-09-12

**Authors:** Ethan Tsai, Brighman Walker, Shiao‐Chi Wu

**Affiliations:** ^1^ Tulane University New Orleans Louisiana USA; ^2^ Asia University Taichung Taiwan; ^3^ National Yang‐Ming Chiao Tung University Taipei Taiwan

**Keywords:** area‐level income, health equity, individual‐level income, late‐stage diagnosis, oral cavity cancer, socioeconomic status

## Abstract

**Background:**

Late‐stage diagnosis of oral cavity cancer (OCC) often results from diagnostic delays due to healthcare access limitations or financial barriers. This study investigates how area‐level (“place”) and individual‐level (“pocketbook”) income influence the stage at OCC diagnosis.

**Methods:**

This retrospective cohort study analyzed data on patients diagnosed with OCC between 2010 and 2013 sourced from Taiwan's National Cancer Registry and National Health Insurance databases. The primary outcome was late‐stage diagnosis, defined as those initially diagnosed at stage III or IV. Multivariable analyses were conducted to estimate the association between area‐level, individual‐level, and late‐stage diagnosis.

**Results:**

This study included 16,652 incident OCC patients, with 6639 (39.9%) diagnosed at a late stage and 10,013 (60.1%) at an early stage. Patients with low individual incomes residing in low‐income areas had 1.48 times higher odds (95% CI = 1.09 to 2.00, *p* = 0.011) of late‐stage diagnosis compared to high‐income patients in high‐income areas. High‐income patients; however, in low‐income areas had 1.34 times higher odds (95% CI = 1.05 to 1.71, *p* = 0.02) to be diagnosed at a late stage compared to high‐income patients in high‐income areas.

**Conclusions:**

While area‐level income plays a significant role in late‐stage OCC diagnosis, higher individual income does not fully protect against late‐stage diagnosis in low‐income areas.

## 1 Introduction

Oral cavity cancer (OCC) represents a substantial global health challenge, with an increasing incidence and disease burden worldwide, particularly in Asia [1, 2]. While the global incidence of OCC is 4.0 per 100,000 people [3], Taiwan has a world‐leading incidence rate of 21.6 cases per 100,000 people [4]. This high OCC incidence in Taiwan is driven by the widespread use of betel nut (also called areca nut), a highly addictive Group 1 carcinogen and risk factor for OCC [5, 6]. Betel nut chewing in Taiwan is embedded in social customs, religious practices, and cultural rituals [7].

Early detection is an essential precursor to effective OCC control. Patients at an advanced stage often deal with more aggressive treatments, significant facial disfigurement, poor prognoses, and diminished quality of life [8, 9, 10]. Unfortunately, approximately 50% of OCC cases are still diagnosed at stage III or IV worldwide [11, 12, 13, 14]. In Taiwan, about 40% of OCC patients are diagnosed at a late stage [15, 16]. Initial diagnosis at a late stage reflects diagnostic delays, often resulting from limited access to healthcare providers or the personal finances necessary to receive care.

The Taiwan National Health Insurance (NHI) system is a single‐payer system that provides universal healthcare coverage and affordable healthcare services. The system is primarily funded through income‐based premiums, guided by the principle of ability to pay, aiming to reduce financial barriers and improve healthcare access [17]. Despite this comprehensive coverage, disparities in late‐stage OCC diagnosis persist.

Existing studies from the United States, Canada, and Denmark indicate that OCC patients living in lower income areas are more likely to receive a late‐stage diagnosis [14, 18, 19, 20] and experience higher mortality rates [21, 22, 23]. Additionally, a cross‐sectional study from India found that lower individual‐level income is associated with diagnostic delays, with delays exceeding 40 days between the first symptoms and a confirmed diagnosis [24]. However, most research on late‐stage OCC diagnosis has primarily focused on area‐level income, without simultaneously examining the role of individual‐level income.

A population‐based longitudinal study in Taiwan found that low‐income OCC patients residing in low‐income areas face significantly higher mortality [25]. However, it remains unclear to what extent these two sources of economic disadvantage—area‐level income (“place”) and individual‐level income (“pocketbook”)—contribute to disparities in OCC late‐stage diagnosis. Therefore, this study aims to disentangle the combined effects of area‐level income and individual‐level income on late‐stage OCC diagnosis in Taiwan.

## 2 Materials and Methods

### 2.1 Study Design and Population

This retrospective cohort study encompassed incident OCC patients (using International Classification of Diseases, 10th Revision code [ICD‐10] codes C00‐C06) diagnosed between January 1, 2010 and December 31, 2013. Data were sourced from the National Cancer Registry database, which accounts for over 97% of OCC patients in Taiwan [16]. A total of 16,897 incident OCC patients with complete cancer stage information were identified in the Cancer Registry database. These cases were then linked to the NHI database, Oral Mucosal Screening database, and Census database to obtain information on OCC screening, demographics, and disease characteristics. After excluding 245 patients with missing individual income, age, marital status, or education, a total of 16,652 incident OCC patients were included in the final analysis.

### 2.2 The Dependent Variable

The study's primary outcome was the late‐stage diagnosis, defined as initial pathological confirmation of stage III or IV by the American Joint Committee on Cancer (AJCC) Cancer Staging Manual 7th Edition. Stages I and II were categorized as early stage. Among all included patients, 99% underwent microscopic examination to complete pathological confirmation [16].

### 2.3 Independent Variables

The independent variables were area‐level income and individual‐level income. Area‐level income was determined based on Taiwan's 368 administrative divisions, which included townships, districts, and county‐administered cities. These divisions serve as the basis for Taiwan's urban–rural classification and are commonly used for policy‐making and epidemiological studies. The average population per division during the study period was approximately 63,000. Area‐level income was determined by the patient's residential area at the time of diagnosis. Patients' zip codes were linked to an open‐source area‐level income database [26] to obtain the annual mean family recurrent income for each administrative division. Area‐level income was categorized into quartiles: low (up to the first income quartile), medium (between the first and third income quartiles), and high (above the third income quartile).

Individual‐level income was defined by the insured enrollee's monthly premium ratable wage reported to the NHI. The monthly premium is calculated based on the insured's actual monthly wage and the number of dependents [17]. For this study, individual‐level income was classified into low (below $582 US dollars [USD]), medium (above $582 but below $1947 USD), and high (above $1947 USD).

### 2.4 Covariates

Covariates included patients' demographics, health behaviors, and disease characteristics. Age was categorized as ≤ 45, 46 to 64, 65 to 84, and ≥ 85. Sex was classified as male or female. Education level was categorized as below college or college and above. Marital status was grouped into unmarried, married, divorced, or widowed. Betel nut chewing, cigarette smoking, and alcohol consumption were categorized as yes, no, quit, and unknown. Oral cancer screening was classified as either yes or no, indicating whether the patient had undergone oral cancer screening prior to the diagnosis date. Comorbidities were measured using the Charlson Comorbidity Index (CCI) to assess the presence of chronic diseases. Primary tumor subsites were categorized as lips (ICD‐10: C00), tongue (ICD‐10: C01‐C02), gum (ICD‐10: C03), floor of mouth (ICD‐10: C04), and palate or other sites (ICD‐10: C05‐C06).

### 2.5 Statistical Analysis

A two‐level hierarchical generalized linear mixed model with random intercepts for areas was applied to assess the association between independent variables and late‐stage diagnosis. Odds ratios (ORs) were estimated using a logit link function for the binomial distribution of late‐stage diagnosis. Multivariable analyses were conducted to calculate adjusted odds ratios (AORs), with all covariates included in the model. Two models were specified in the analysis. The first model included individual‐level income, area‐level income, and all covariates in order to estimate their main effect on late‐stage diagnosis. The second model extended the analysis by including an interaction term between individual‐level and area‐level income to evaluate whether the effect of individual‐level income varied by area‐level income strata. Both models adjusted for age, sex, education, marital status, past oral cancer screening, chewing betel nuts, smoking cigarettes, drinking alcohol, comorbidity, and primary subsites. Collinearity was assessed using the Variance Inflation Factor (VIF), with a maximum VIF of 1.096, indicating no multicollinearity among the variables. All data management and statistical analyses were conducted using SAS version 9.4.

## 3 Results

A total of 16,652 OCC patients were included in this study. Of these patients, 40.0% were diagnosed with late‐stage OCC, 66.8% resided in a medium‐income area, and 56.9% had a medium individual income. The majority of OCC patients were male (91.0%), aged 46 to 64 years (66.8%), married (72.1%), had an education level below college (90.3%), had undergone oral cancer screening (58.2%), smoked cigarettes (36.8%), consumed alcohol (33.4%), chewed betel nut (31.0%), had comorbidities (51.5%), and had primary subsites of the palate and others (43.4%) (Table 1).

**TABLE 1:** Characteristics of oral cavity cancer patients.

	*N* (%)	Late stage (%)	Early stage (%)
Total	16,897 (100)	6766 (100)	10,131 (100)
Area‐level income			
Low	3062 (18.12)	1231 (18.19)	1831 (18.07)
Medium	11,284 (66.78)	4482 (66.24)	3802 (37.53)
High	2551 (15.10)	1053 (15.56)	1498 (14.79)
Individual‐level income			
Low	4640 (27.46)	2094 (30.95)	2546 (25.13)
Medium	9607 (58.86)	3715 (54.91)	5892 (58.16)
High	2405 (14.23)	830 (12.27)	1575 (15.55)
Missing	245 (1.45)	127 (1.88)	118 (1.16)
Age			
≤ 45	11,290 (66.82)	3733 (55.17)	7557 (74.59)
46–64	3478 (20.58)	2028 (29.97)	1450 (14.31)
65–84	1775 (10.50)	857 (12.67)	918 (9.06)
≥ 85	149 (0.88)	43 (0.64)	106 (1.05)
Missing	205 (1.21)	105 (1.55)	100 (0.99)
Sex			
Male	15,379 (91.02)	6255 (92.45)	9124 (90.06)
Female	1518 (8.98)	511 (7.55)	1007 (9.94)
Marital status			
Unmarried	1596 (9.45)	737 (10.89)	859 (8.48)
Married	12,176 (72.06)	4694 (69.38)	7482 (73.85)
Divorced	2278 (13.48)	989 (14.62)	1289 (12.72)
Widowed	844 (4.99)	346 (5.11)	498 (4.92)
Missing	3 (0.02)	0 (0)	3 (0.03)
Education			
Below college	15,263 (90.33)	6183 (91.38)	9080 (89.63)
Above college	1628 (9.63)	582 (8.6)	1046 (10.32)
Missing	6 (0.04)	1 (0.01)	5 (0.05)
Oral cancer screening			
No	7068 (41.83)	2995 (44.27)	4073 (40.2)
Yes	9829 (58.17)	3771 (55.73)	6058 (59.8)
Chewed betel nut			
No	3997 (23.66)	1404 (20.75)	2593 (25.59)
Quit	1702 (10.07)	646 (9.55)	1056 (10.42)
Yes	5242 (31.02)	2211 (32.68)	3031 (29.92)
Unknown	5956 (35.25)	2505 (37.02)	3451 (34.06)
Smoked cigarettes			
No	2637 (15.61)	894 (13.21)	1743 (17.2)
Quit	1044 (6.18)	387 (5.72)	657 (6.49)
Yes	6212 (36.76)	2585 (38.21)	3627 (35.8)
Unknown	7004 (41.45)	2900 (42.86)	4104 (40.51)
Drank alcohol			
No	4434 (26.24)	1586 (23.44)	2848 (28.11)
Quit	2468 (14.61)	1052 (15.55)	1416 (13.98)
Yes	5639 (33.37)	2349 (34.72)	3290 (32.47)
Unknown	4356 (25.78)	1779 (26.29)	2577 (25.44)
Comorbidity			
CCI = 0	81,99 (48.52)	3582 (52.94)	4617 (45.57)
CCI = 1	1715 (10.15)	587 (8.68)	1128 (11.13)
CCI = 2	4198 (24.84)	1541 (22.78)	2657 (26.23)
CCI = 3	1400 (8.29)	485 (7.17)	915 (9.03)
CCI > =4	1385 (8.2)	571 (8.44)	814 (8.03)
Primary subsite			
Tongue	5816 (34.42)	2131 (31.5)	3685 (36.37)
Lips	1061 (6.28)	277 (4.09)	784 (7.74)
Gum	2096 (12.4)	1329 (19.64)	767 (7.57)
Floor of mouth	570 (3.37)	219 (3.24)	351 (3.46)
Palate and others	7354 (43.52)	2860 (42.27)	4494 (44.36)

Abbreviation: CCI, Charlson Comorbidity Index.

Compared with patients diagnosed at an early stage, those diagnosed at a late stage were more likely to be younger than 45 years (55.2% vs. 74.6%), male (92.5% vs. 90.0%), unmarried (10.9% vs. 8.5%) or divorced (14.6% vs. 12.7%), and to have an education below college level (91.4% vs. 89.6%). They were also more likely to report betel nut chewing (32.7% vs. 29.9%), alcohol consumption (34.7% vs. 32.5%), and cigarette smoking (38.2% vs. 35.8%). By contrast, patients diagnosed at an early stage were more likely to have undergone oral cancer screening (59.8% vs. 55.7%) (Table 1).

While area‐level income was not statistically significantly associated with late‐stage diagnosis, individual income was: Patients with medium individual incomes (AOR = 1.12, 95% CI = 1.02 to 1.25, *p* = 0.02) or low individual incomes (AOR = 1.45, 95% CI = 1.30 to 1.63, *p* < 0.01) had a statistically significantly higher odds of late‐stage diagnosis than high‐income patients (Table 2). Estimates for all covariates in relation to late‐stage diagnosis are also presented in Table 2.

**TABLE 2:** Association between area‐level income or individual‐level income and late‐stage diagnosis.

	Unadjusted model			Adjusted model		
	COR	95% CI	*p*	AOR	95% CI	*p*
Area‐level income						
High (reference)						
Medium	0.96	(0.86–1.06)	0.77	0.83	(0.67–1.02)	0.10
Low	0.94	(0.86–1.02)	0.20	0.88	(0.74–1.06)	0.16
Individual‐level income						
High (reference)						
Medium	1.20	(1.09–1.32)	< 0.01	1.12	(1.02–1.25)	0.02
Low	1.57	(1.41–1.74)	< 0.01	1.45	(1.30–1.63)	< 0.01
Age						
≤ 45 (ref)						
46–64	2.92	(2.70–3.15)	< 0.01	2.98	(2.74–3.24)	< 0.01
65–84	1.97	(1.97–2.17)	< 0.01	2.20	(1.97–2.46)	< 0.01
≥ 85	0.84	(0.59–1.91)	0.83	0.96	(0.65–1.40)	0.81
Sex						
Male (ref)						
Female	0.75	(0.67–0.84)	< 0.01	0.88	(0.77–1.01)	0.07
Education						
Above college (ref)						
Below college	0.82	(0.74–0.91)	< 0.01	0.87	(0.78–0.98)	0.02
Marital status						
Unmarried (ref)						
Married	0.73	(0.66–0.81)	< 0.01	0.75	(0.66–0.84)	< 0.01
Divorce	0.89	(0.79–1.02)	0.22	0.79	(0.69–0.91)	< 0.01
Widowed	0.81	(0.68–0.96)	0.35	0.88	(0.73–1.07)	0.21
Oral cancer screening						
No (ref)						
Yes	1.02	(0.96–1.09)	0.46	1.00	(0.93–1.07)	0.99
Chewing betel nuts						
No (ref)						
Quitted	1.15	(1.03–1.29)	0.50	0.99	(0.86–1.14)	0.87
Yes	1.35	(1.24–1.47)	< 0.01	1.19	(1.06–1.32)	< 0.01
Unknown	1.33	(1.23–1.44)	< 0.01	1.32	(1.15–1.51)	< 0.01
Smoked cigarette						
No (ref)						
Quitted	1.16	(1.00–1.35)	0.37	0.99	(0.82–1.19)	0.89
Yes	1.38	(1.26–1.52)	< 0.01	1.14	(1.01–1.30)	0.03
Unknown	1.35	(1.23–1.48)	< 0.01	1.14	(1.00–1.32)	0.06
Drank alcohol						
No (ref)						
Quit	1.35	(1.22–1.49)	< 0.01	1.15	(1.02–1.29)	0.02
Yes	1.29	(1.19–1.39)	< 0.01	1.10	(1.00–1.22)	0.06
Unknown	1.25	(1.12–1.32)	0.71	0.88	(0.75–1.02)	0.09
Comorbidity						
CCI = 0 (ref)						
CCI = 1	0.68	(0.61–0.76)	< 0.01	0.68	(0.60–0.76)	< 0.01
CCI = 2	0.76	(0.71–0.82)	0.09	0.67	(0.62–0.73)	< 0.01
CCI = 3	0.69	(0.62–0.78)	< 0.01	0.59	(0.52–0.68)	< 0.01
CCI > =4	0.93	(0.83–1.04)	< 0.01	0.74	(0.65–0.84)	< 0.01
Primary subsite						
Tongue (ref)						
Lip	0.48	(0.41–0.56)	< 0.01	0.44	(0.37–0.52)	< 0.01
Gum	3.00	(2.71–3.33)	< 0.01	3.15	(0.28–3.52)	< 0.01
Floor of mouth	1.10	(0.93–1.31)	0.27	0.97	(0.81–1.17)	0.77
Palate and others	1.10	(1.03–1.18)	< 0.01	1.06	(0.98–1.14)	0.15

*Note:* *N* = 16,652.

Abbreviations: AOR, adjusted odds ratio; CCI, Charlson Comorbidity Index; COR, Crude odds ratio.

However, area environments also appear to matter as individual incomes were only associated with statistically significant differences in low‐income areas. Compared to high‐income patients in high‐income areas, those with low individual incomes in low‐income areas were 1.48 times (95% CI = 1.09 to 2.00, *p* = 0.01) more likely to have a late‐stage diagnosis. Even high‐income patients in low‐income areas were 1.34 times (95% CI = 1.05 to 1.71, *p* = 0.02) more likely to have a late‐stage diagnosis (Table 3).

**TABLE 3:** The combined association between area‐level income or individual‐level income and late‐stage diagnosis.

		Area‐level income											
		Low				Medium				High			
		*N* (%)	AOR	95% CI	*p*	*N* (%)	AOR	95% CI	*p*	*N* (%)	AOR	95% CI	*p*
Individual‐level Income	Low	572 (3.39)	1.48	(1.09–2.00)	0.01	3257 (19.28)	0.98	(0.75–1.28)	0.88	811 (4.80)	0.83	(0.58–1.19)	0.31
	Medium	2192 (12.97)	1.27	(0.99–1.63)	0.06	6209 (36.75)	1.00	(0.78–1.28)	0.98	1206 (7.14)	1.12	(0.89–1.41)	0.33
	High	266 (1.57)	1.34	(1.05–1.71)	0.02	1647 (9.75)	1.12	(0.89–1.41)	0.33	492 (2.91)	(ref)	—	—

*Note:* *N* = 16,652. This model was adjusted by age, sex, education, marital status, past oral cancer screening, chewing betel nuts, smoking cigarettes, drinking alcohol, comorbidity, and primary subsites.

Abbreviation: AOR, adjusted odds ratio.

## 4 Discussion

Our findings underscore the importance of income at both area‐level and individual‐level in relation to late‐stage OCC diagnosis: low individual income does not appear to drive late‐stage OCC diagnosis among those living in high‐income areas and, conversely, a high individual income does not appear to meaningfully protect against the likelihood of late‐stage OCC diagnosis among those living in low‐income areas. Although we did not find a statistically significant association between area‐level income and late‐stage diagnosis, which does not align with previous studies from the United States, Canada, and Denmark [14, 18, 19, 20], our results highlight the distinct roles of area‐level and individual‐level socioeconomic status (SES) in influencing late‐stage diagnosis.

A population‐based longitudinal study in Taiwan found SES disparities in OCC mortality rates at both the individual and area levels, with the combined effect of low individual‐level income and low area‐level SES posing the highest risk of death [25]. Our findings offer a potential explanation for this higher risk, as late‐stage diagnosis could contribute to poorer survival outcomes among these patients.

This study provides valuable evidence on health disparities related to area‐level and individual‐level income in late‐stage OCC diagnosis in a high‐incidence country, despite Taiwan's lower financial barriers to care under its NHI program. Since individual‐level financial barriers are minimized within this system, the disparities observed at the area level are more likely to reflect differences in access to care. Policy interventions are needed to mitigate income‐based disparities in late‐stage OCC diagnosis.

Taiwan implemented a nationwide oral cancer screening program in 2004, and in our study, 58% of patients had undergone screening. However, our findings indicate that patients in low‐income areas—even those with high individual income—are more likely to be diagnosed at a late stage, suggesting potential barriers to timely access to care. Therefore, increasing oral cancer screening rates in low‐income areas, coupled with allocating additional resources for case management and follow‐up of individuals identified through screening, could help facilitate the early detection of cancer symptoms, timely referral, and diagnostic confirmation for OCC patients in low‐income areas. Health education initiatives focusing on early symptom recognition could also reduce diagnostic delays in low‐income areas.

Although these results may not be generalizable to countries without universal health coverage, this study provides valuable insights from a high‐incidence country with a well‐established NHI system and relatively low financial barriers. Additionally, recent studies raising concerns about the growing availability of betel nut within Asian communities further exacerbate the risk of late‐stage diagnosis in the United States [27, 28]. Our findings may be relevant for policymakers in other countries with rising OCC incidence to understand and address income‐based disparities in healthcare access and outcomes.

To our knowledge, this is the first study to evaluate the combined effects of area‐level and individual‐level income on late‐stage OCC diagnosis. Using a large nationally representative dataset, this study provides findings that can be generalized to the Taiwanese OCC population. Moreover, it offers policymakers evidence to develop targeted strategies aimed at reducing late‐stage OCC diagnosis and improving health equity.

### 4.1 Limitations

The primary limitation of this study is that the data analyzed were from 2010 to 2013, as these were the most recent complete datasets available at the time of analysis. However, Taiwan's NHI system and oral cancer screening program have remained largely consistent during this period, and Taiwan continues to have one of the highest OCC incidence rates worldwide; therefore, we believe our findings remain informative and relevant. Additionally, this study lacks data to investigate some potential reasons behind late‐stage diagnosis, such as distance to healthcare facilities, health literacy, and cultural factors. We also did not have information on Human Papillomavirus (HPV), an important risk factor for OCC. Furthermore, the dataset did not allow us to determine whether patients had changed their residence, which could affect area‐level SES classification.

## 5 Conclusion

This study highlights inequities in OCC diagnosis related to both area‐level and individual‐level income. Notably, higher individual income does not fully protect against late‐stage diagnosis in low‐income areas, indicating distinct roles of area‐level and individual‐level SES influences. These results underscore the urgency for additional policies and interventions to alleviate income‐based disparities in oral cancer care.

## Author Contributions

**Ethan Tsai:** conceptualization (lead), formal analysis (lead), investigation (lead), methodology (lead), software (lead), visualization (lead), writing – original draft (lead). **Brighman Walker:** conceptualization (equal), methodology (equal), validation (equal), writing – review and editing (lead). **Shiao‐Chi Wu:** data curation (lead), funding acquisition (lead), project administration (lead), resources (lead), supervision (lead), validation (lead).

## Ethics Statement

This study was approved by the Institutional Review Board of National Yang‐Ming University (IRB approval number: 1070601–2).

## Consent

The requirement for informed consent was waived by the Institutional Review Board of National Yang‐Ming University (IRB approval number: 1070601–2) due to the retrospective nature of the study and the use of de‐identified data.

## Conflicts of Interest

The authors declare no conflicts of interest.

## Data Availability

Data sharing is not applicable to this article as all data are managed by Taiwan's Ministry of Health and Welfare and are accessible exclusively at the Center of Health and Welfare Data Science.
